# Blockade of TMPRSS2-mediated priming of SARS-CoV-2 by lactoferricin

**DOI:** 10.3389/fimmu.2022.958581

**Published:** 2022-08-23

**Authors:** Anna Ohradanova-Repic, Rostislav Skrabana, Laura Gebetsberger, Gabor Tajti, Peter Baráth, Gabriela Ondrovičová, Romana Praženicová, Nikola Jantova, Patricia Hrasnova, Hannes Stockinger, Vladimir Leksa

**Affiliations:** ^1^ Medical University of Vienna, Center for Pathophysiology, Infectiology and Immunology, Institute for Hygiene and Applied Immunology, Vienna, Austria; ^2^ Laboratory of Structural Biology of Neurodegeneration, Institute of Neuroimmunology, Slovak Academy of Sciences, Bratislava, Slovakia; ^3^ Department of Glycobiology, Institute of Chemistry, Slovak Academy of Sciences, Bratislava, Slovakia; ^4^ Laboratory of Molecular Immunology, Institute of Molecular Biology, Slovak Academy of Sciences, Bratislava, Slovakia; ^5^ Department of Biochemistry, Comenius University, Bratislava, Slovakia

**Keywords:** COVID-19, SARS-CoV-2, coronavirus, virus priming, serine proteases, TMPRSS2, lactoferrin, lactoferricin

## Abstract

In addition to vaccines, there is an urgent need for supplemental antiviral therapeutics to dampen the persistent COVID-19 pandemic caused by the severe acute respiratory syndrome coronavirus-2 (SARS-CoV-2). The transmembrane protease serine 2 (TMPRSS2), that is responsible for proteolytic priming of the SARS-CoV-2 spike protein, appears as a rational therapeutic target. Accordingly, selective inhibitors of TMPRSS2 represent potential tools for prevention and treatment of COVID-19. Previously, we identified the human milk glycoprotein lactoferrin as a natural inhibitor of plasminogen conversion to plasmin, a serine protease homologous to TMPRSS2. Here, we tested whether lactoferrin and lactoferricin, a biologically active natural peptide produced by pepsin-mediated digestion of lactoferrin, together with synthetic peptides derived from lactoferrin, were able to block TMPRSS2 and SARS-CoV-2 infection. Particularly, we revealed that both lactoferricin and the N-terminal synthetic peptide pLF1 significantly inhibited: i) proteolytic activity of TMPRSS2 and plasmin, ii) proteolytic processing of the SARS-CoV-2 spike protein, and iii) SARS-CoV-2 infection of SARS-CoV-2-permissive cells. Thus, natural and synthetic peptides derived from lactoferrin represent feasible candidates for supporting prevention and treatment of COVID-19.

## Introduction

The global COVID-19 pandemic is unceasingly firing up a demand for cheap and available therapeutics to supplement standard treatment protocols. The angiotensin converting enzyme-2 (ACE2) serves as a dominant host cell-surface receptor for the severe acute respiratory syndrome coronavirus-2 (SARS-CoV-2) through engagement of the viral spike (S) protein (1, 2), although additional interaction sites in virus-cell attachment have been suggested, including heparan sulfate proteoglycans (HSPGs) (3). Upon binding, proteolytic processing of the S protein, i.e. virus priming (4, 5), by host serine proteases, in particular by the transmembrane protease serine 2 (TMPRSS2) (6), is essential for fusion of viral and cellular membranes, and subsequent virus entry to the host cell.

Colocalization of ACE2 and TMPRSS2, a canonical priming protease of both SARS-CoV-1 and SARS-CoV-2, is crucial for viral infection of target cells. S protein interacts with ACE2 *via* its N-terminal moiety (S1) encompassing the receptor-binding domain (RBD), and then is cleaved by proximal TMPRSS2 at the site between S1 and the C-terminal moiety (S2). This initial cleavage is followed by cleavage at the S2’ site, which reveals the Ser816-Phe833 fusion peptide and triggers a fusion of viral membrane with the host cell membrane (4, 5). Thus, the inhibition of each event, virus binding, priming and fusion, represents a promising piece in the puzzle of managing the COVID-19 pandemic (7–9).

Active sites of several human serine proteases exhibit high identity with TMPRSS2, the one of plasminogen even 95% (10). Previously, we identified the human milk glycoprotein lactoferrin (LF) as an inhibitor of the proteolytic conversion of plasminogen to plasmin (11). Here, we expand these data to TMPRSS2. In addition to full-length LF, we tested its N-terminal bioactive peptide called lactoferricin (LFC), which is known for its antiviral and antibacterial activities. LFC is physiologically produced from the N-terminal region of LF upon cleavage by pepsin (12, 13). In a similar way, we produced LFC *in vitro*. We show that LFC and the corresponding synthetic peptide pLF1 designed by us from the N-terminal region of LF inhibit TMPRSS2, and subsequently, SARS-CoV-2 priming and infection of target cells.

## Results

Previously, we described the binding of lactoferrin (LF) to plasminogen (Plg), which blocked Plg conversion to the active serine protease plasmin. According to our mapping studies, LF bound to Plg *via* its highly cationic N-terminal region (11). Since structures of the catalytic domains of serine proteases display a high level of identity (10), we have tested the inhibitory effect of LF and three LF-derived synthetic peptides against other serine proteases, including TMPRSS2, elastase and trypsin, in addition to plasmin. The peptides we used were as follows: pLF1 from the N-terminal region, pLF2 from the helix linker region, and pLF3 from the C-terminal region (11). Additionally, we used peptide pCTR as a negative control (11) and the broad-spectrum serine protease inhibitor aprotinin as a positive control.

**Figure 1:**
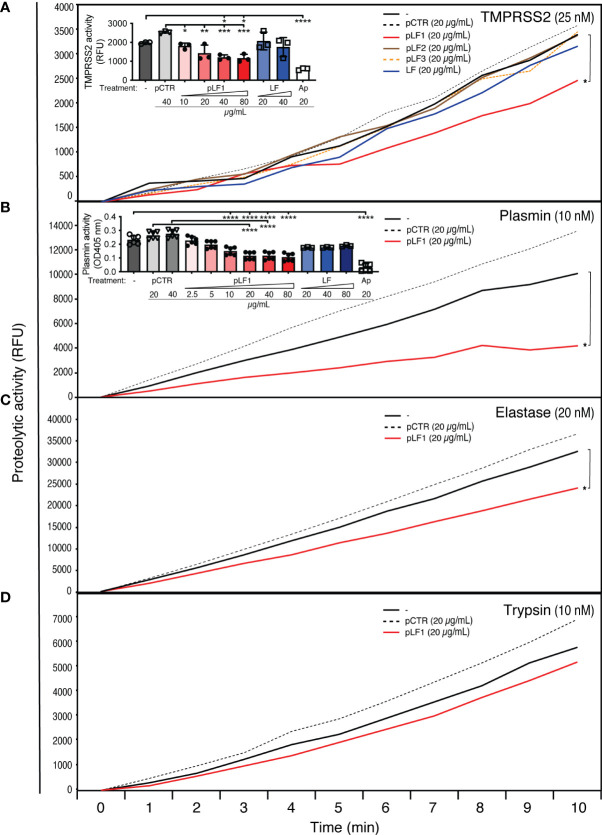
Effect of LF and the LF-derived peptides pLF1-pLF3 on the activity of serine proteases. Proteolytic activities of purified TMPRSS2 **(A)**, plasmin **(B)**, elastase **(C)**, and trypsin **(D)** were measured with or without peptides pLF1, pLF2, pLF3, LF or the control peptide pCTR at 37°C in Tris-HCl buffer using the fluorogenic substrate Boc-Gln-Ala-Arg-AMC. The reaction was monitored using an ELISA reader for the indicated time intervals. The *insets* in **(A)** and **(B)** show concentration-dependent inhibition of TMPRSS2 and plasmin activities by pLF1 but not by LF after 30 min and 4 h, Values of p*<0.05, p**<0.005, p***<0.0005, p****<0.0001 (as indicated) were considered to be significant or highly significant, respectively.

**Figure 2:**
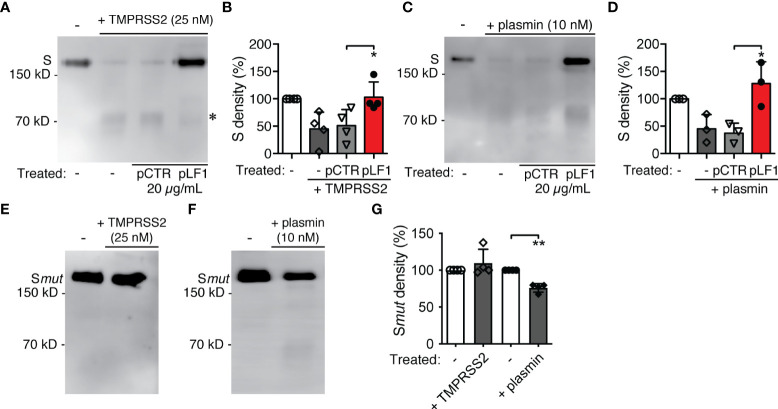
Effect of LF and the LF-derived peptides on SARS-CoV-2 S protein proteolytic processing. **(A)** The purified recombinant 6x-His-tagged S protein was exposed to TMPRSS2 with or without pLF1 or pCTR at 37°C in Tris-HCl buffer for 2 h. The digestion of the S protein was analyzed by immunoblotting of the reaction mixtures followed by an incubation with the specific anti-6x-His mAb. Upon cleavage by TMPRSS2, a digestion product of the S protein of about 75 kD became visible (marked by *). **(B)** Densitometric quantifications of the proteolytic processing of the S protein by TMPRSS2. Peptide pCTR served as a control. Four independent immunoblotting experiments were evaluated. The bands corresponding to the full-length S protein in the absence of TMPRSS2 were set as a relative maximum of 100%. The immunoblots were quantified by the AzureSpot software. **(C)** Purified recombinant 6x-His-tagged S protein was exposed to plasmin instead of TMPRSS2 and treated afterwards as in **(A)**. **(D)** Densitometric quantifications of the proteolytic processing of the S protein by plasmin. Peptide pCTR served as a control. Densitometric evaluation of three independent immunoblotting experiments was performed as in **(B)**. **(E-G)** The purified recombinant 8x-His-tagged mutated S protein was treated in *in vitro* proteolysis assay with TMPRSS2 **(E)** and plasmin **(F)** as in **(A, C)**, and the cleavage **(G)** was evaluated as in **(B, D)** Values of p*<0.05, p**<0.005 (as indicated) were considered to be significant..

An *in vitro* proteolysis assay at 37°C using the fluorogenic serine protease substrate Boc-Gln-Ala-Arg-AMC revealed that pLF1, but neither full-length LF nor other tested peptides derived from LF, nor the control peptide pCTR, significantly reduced the proteolytic activity of TMPRSS2 (**Figure 1A**), plasmin or elastase (**Figures 1B, C**). The inhibitory effect of pLF1 on trypsin was not significant (**Figure 1D**). pLF1 inhibited TMPRSS2 with the half-maximum inhibitory concentration (IC50) of 9.8 µg/mL, with maximum inhibition at 40 - 80 µg/mL, approaching a plateau of ~ 40% inhibition (**Figure 1A**, *inset*). For plasmin, the IC50 was 6.9 µg/mL, with maximum inhibition observed at 20 µg/mL (**Figure 1B**, *inset*). In contrast, full-length LF exhibited no dose-dependent inhibition of either TMPRSS2 or active plasmin (**Figures 1A, B**, *insets*). The competitive inhibitor aprotinin blunted the proteolytic activity of both proteases (**Figures 1A, B**, *insets*). Thus, while LF apparently blocked only the conversion of inactive Plg to the active serine protease plasmin (11), the N-terminal peptide pLF1 is able to execute its inhibitory effect on already active TMPRSS2, plasmin, and elastase, which implies that pLF1 may directly target the catalytic domain of serine proteases**Figures 2D**.

TMPRSS2 was shown to process the S protein of the pathogenic coronaviruses SARS-CoV-1 and SARS-CoV-2 and, as a consequence, to play a central role in the coronavirus entry into a host cell (14–17). When we exposed the recombinant SARS-CoV-2 S protein to TMPRSS2 *in vitro*, we found that the S protein was cleaved by TMPRSS2 to a diffuse fragment of about 75 kD (**Figure 2A**) that agreed with the molecular size of the S2 domain of SARS-CoV-2 (18, 19). When we added pLF1 to the reaction, the cleavage was blocked; no such effect was seen with pCTR (**Figures 2A, B**). The incubation of the S protein with plasmin produced more bands within this size range, which might indicate that processing by plasmin was not as specific as by TMPRSS2. The cleavage by plasmin was also hindered in the presence of pLF1 (**Figures 2C, D**). When we performed the *in vitro* proteolysis assay with the S protein mutated at the S1/S2 site, we did not observe any cleavage by TMPRSS2 and only a minor cleavage with plasmin, which further supported the assumption that plasmin was less specific (**Figures 2E–G**). Together, these data indicate that the N-terminal fragment of LF can inhibit the proteolytic processing of the SARS-CoV-2 S protein.

**Figure 3:**
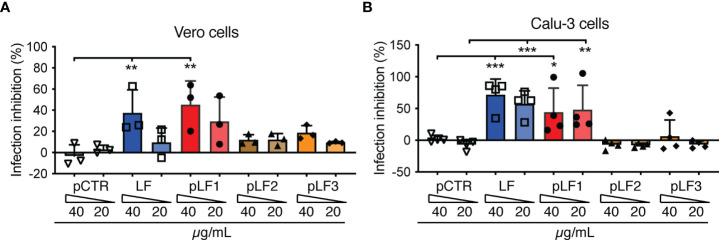
Effect of LF and the LF-derived peptides pLF1-pLF3 on the infection capability of SARS-CoV-2. Vero cells **(A)** or Calu-3 cells **(B)** grown in 96-well plates were incubated with or without LF, peptides pLF1, pLF2, pLF3 or pCTR for approx. 1 h and then infected with the authentic SARS-CoV-2 (300 TCID50/well). After 48 h, the cells were fixed and the infection rate was assessed by In-Cell ELISA. Values show mean inhibitory capacity of LF and the indicated peptides ± SD from three **(A)** or four **(B)** independent experiments. p*<0.05, p**<0.005, p***<0.0005. Significance was evaluated by two-way ANOVA with Dunnett’s multiple comparisons post-test.

To test the capacity of LF or LF-derived peptides to inhibit SARS-CoV-2 infection, we selected Vero cells, which, in contrast to Vero E6 cells, express detectable amounts of TMPRSS2 (20). Before replication-competent SARS-CoV-2 was added, the Vero cells were pre-incubated with LF, the LF-derived peptides or control pCTR for 40-60 min. As shown in **Figure 3A**, the N-terminal peptide pLF1 significantly reduced SARS-CoV-2 infection by ≈40% when used at 40 µg/mL, while the other two peptides failed to do so, although some non-significant reduction was observed at 40 µg/mL (**Figure 3A**). pLF1 also showed some inhibitory potential at 20 µg/mL (≈30% inhibition), but the differences were not significant. The control peptide pCTR did not show any inhibitory potential. Of note, the peptides did not show any cytotoxic effects when incubated with Vero cells in the absence of the virus (**Supplementary Figure 1**).

When we repeated the assay with the human lung cell line Calu-3 that highly expresses TMPRSS2 (6), from the peptides tested, only pLF1 was able to significantly inhibit SARS-CoV-2 infection by ≈45% at both concentrations used (**Figure 3B**). Interestingly, although full-length LF was not able to block TMPRSS2 activity (**Figure 1A**), it prevented infection of Vero cells by ≈38% when used at 40 µg/mL (**Figure 3A**), and seemed even more potent in Calu-3 cells, where ≈60-70% inhibition was observed (**Figure 3B**). This might be likely attributed to LF-mediated blockade of cell-surface HSPGs that aid coronavirus infection (3). Hence, LF seems to obstruct the cell entry of SARS-CoV-2 *via* at least two independent mechanisms.

**Figure 4:**
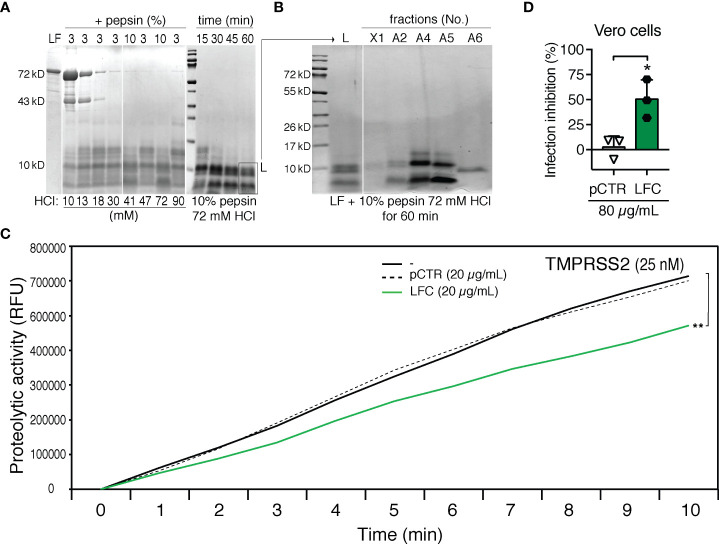
Generation and testing of human LFC. **(A)** Optimizations of HCl and pepsin concentrations (mM and % (w/w), respectively) on LF cleavage. LF (10.5 mg/mL) was exposed to pepsin (3% or 10% per amount of LF) and HCl (10-90 mM, as indicated) for 30 min (left panel) or various time points (15, 30, 45 or 60 min, right panel) at 37°C. Digestion mixtures were analyzed by 10% tricine-SDS-PAGE and subjected to Coomassie blue staining. **(B)** Separation of LF cleavage products by cation exchange chromatography. Individual fractions were analyzed by 10% tricine-SDS-PAGE and subjected to Coomassie blue staining: L – digestion mixture, X1 - material not captured on the column, A2, A4, A5, A6 - eluted fractions. **(C)** Effect of LFC (fraction A6) on the activity of TMPRSS2. Proteolytic activity of TMPRSS2 was measured with or without LFC or pCTR at 37°C in Tris-HCl buffer using the fluorogenic substrate Boc-Gln-Ala-Arg-AMC. The reaction was monitored using an ELISA reader for the indicated time intervals. **(D)** Effect of LFC on the infection capability of SARS-CoV-2. Vero cells were incubated with or without LFC or pCTR (both 80 µg/mL) for approx. 1 h, and then infected with the authentic SARS-CoV-2 (300 TCID50/well; MOI ≈0.02). After 48 h, the cells were fixed and the infection rate was assessed by In-Cell ELISA. Values show mean inhibitory capacity of LFC and pCTR ± SD from three independent experiments. p*<0.05.

Biologically active natural peptides, called lactoferricins (LFCs), with antiviral and antibacterial activities are physiologically produced from the N-terminal region of LF upon cleavage by pepsin (12, 13). Therefore, we tested whether LFCs would display a similar inhibitory effect towards TMPRSS2 as the synthetic peptide pLF1. To yield LFC from intact recombinant human LF, we optimized the cleavage conditions (**Figure 4A**) and chose 60 min incubation in 72 mM HCl and 10% pepsin (per amount of LF) for the subsequent purification. Due to the strong positive charge of LFCs, we used cation exchange chromatography (**Figure 4B**). The fraction A6, eluted at relatively high conductivity (73-81 mS/cm), was supposed to contain the peptide with the highest isoelectric point. A prominent peptide with an apparent molecular size below 10 kD present in fraction A6 was then analyzed by MALDI-TOF MS. Predominant ions present in spectra corresponded to a peptide with the neutral mass of 5738.1 Da, which was in a good agreement with the published peptide mass of the human LFC spanning 49 amino acids from the N-terminus of mature human LF (21).

An *in vitro* proteolytic activation assay with the fluorogenic substrate Boc-Gln-Ala-Arg-AMC showed that the isolated LFC displayed an inhibitory effect on TMPRSS2 comparable to that of pLF1 (**Figure 4C**). Accordingly, the isolated LFC reduced the infection rate of replication-competent SARS-CoV-2 in Vero cells (**Figure 4D**).

Taken together, our data strongly indicate that the N-terminal peptide fragment of LF, LFC, inhibits cell entry of SARS-CoV-2 through blocking TMPRSS2-mediated virus priming.

## Discussion

As the global threat of the COVID-19 pandemic persists, it becomes necessary to complement standard treatment protocols and support a vaccine campaign with new causative therapeutics. Virus priming by TMPRSS2 is crucial for the cell entry of SARS-CoV-2, and represents a promising therapeutic target.

Here, we show that the N-terminus of LF contributes to the defense against SARS-CoV-2 infection *via* blockade of TMPRSS2: both the N-terminal synthetic peptide (designed by us and termed pLF1) and the natural N-terminal peptide, produced by controlled pepsin cleavage of LF, inhibit the proteolytic priming of the viral spike protein by TMPRSS2, and subsequently, cell infection by SARS-CoV-2.

We previously found that full-length LF was able to inhibit activation of Plg through hindering the action of urokinase-type plasminogen activator (uPA), a Plg activator. In contrast, uPA-independent Plg conversion by the bacterial enzyme streptokinase was not affected by LF (11). According to our mapping studies, LF binds to Plg *via* its highly cationic N-terminal region, corresponding to LFC and pLF1 (11). We show that pLF1, but neither full-length LF nor a control peptide (pCTR), was able to inhibit even the intrinsic proteolytic activity of active plasmin. Similarly, only pLF1 but neither full-length LF nor pCTR inhibited the proteolytic activity of closely related TMPRSS2. These findings imply that LF and its N-terminal fragment exploit different inhibitory mechanisms, while LF blocks conversion of inactive Plg to plasmin, the N-terminal peptide pLF1 displays its protease inhibitory effect directly on active plasmin and TMPRSS2. The concentration dependence of inhibition suggests a non-competitive inhibitory mechanism whereby the binding of pLF1 to these active proteases might allosterically decrease the enzyme catalytic rate.

The human glycoprotein LF is synthetized by exocrine glands and is present at high concentrations in secretory body fluids, particularly in milk, but also within inflammatory granules of neutrophils. From the beginning of the pandemic, LF has been recognized as a potential drug candidate (22, 23), and recently, it was found to inhibit SARS-CoV-2 infection *in vitro* (24). Moreover, in a clinical study, supplementary LF treatment significantly reduced COVID-19 symptoms (25). This effect was mainly attributed to its immunomodulatory properties (26).

In our experiments, LF did not block TMPRSS2 activity, yet it prevented SARS-CoV-2 infection. This effect thus might be due to the LF-mediated blockade of HSPGs, since previous research revealed that LF inhibited the SARS-CoV-1 infection through blocking the interaction between the S protein and HSPGs (3). Interestingly, the LF binding to both heparin and HSPGs was observed previously, and it was proposed that the N-terminal region of LF was involved in the binding, thereby inhibiting cell infection by several human coronaviruses, including SARS-CoV-2 (27, 28). Hence, LF and its derived bioactive peptides seem to hinder the cell entry of SARS-CoV-2 through various mechanisms. Additionally, LF appears to inhibit SARS-CoV-2 also at the post-cell entry stage, since it was reported that LF treatment led to e. g. enhancement of interferon responses (24), inhibition of viral replication (29), or maintenance of iron homeostasis (30). Whether the N-terminal region of LF shares some of these antiviral mechanisms with full-length LF, remains to be determined in future studies.

Several biologically active natural peptides derived from the N-terminus of both bovine and human LF by pepsin cleavage, termed lactoferricins (LFCs), were described (13, 21, 31). LFCs possess antibacterial and antiviral activities (32) and their putative activity against SARS-CoV-2 was predicted by simulation (33). Here we report that human LFC as well as the shorter pLF1 peptide from the N-terminal LF region corresponding to LFC, were able to inhibit SARS-CoV-2 infection in Vero and Calu-3 cells to around 40-50%. It has been reported that SARS-CoV-2 can employ in both cell lines also an endosomal pathway for entry, wherein the SARS-CoV-2 S protein is primed by the cysteine protease cathepsin L (6, 15, 34, 35), which is likely the reason why we have not observed full virus inhibition by the LF-derived N-terminal peptides.

On an equimolar level, the full-length LF may be more potent than peptides. The differences between full-length LF and LF-derived peptides may be attributed to the changes in the peptide secondary structures after their release from the compact LF molecule so that it is not possible for peptides to fold into the original conformation (36). Notably, the intestinal mucosa was shown to contain ACE2 and TMPRSS2 molecules, therefore it is not surprising that SARS-CoV-2 efficiently infects gastrointestinal tissue (37, 38). On this basis, the LFC-mediated inhibition of SARS-CoV-2 infection may be of special relevance under intestinal proteolytic conditions wherein the intact LF is not present.

In addition to TMPRSS2, also other host serine proteases were proposed as therapeutic targets to block SARS-CoV-2 cell entry (4, 5, 8, 9, 39, 40). Here, we show that peptide pLF1 derived from the N-terminus of LF inhibits processing of the S protein not only by TMPRSS2 but also by plasmin. Notably, a high plasmin level is considered as risk factor for development of COVID-19 after SARS-CoV-2 infection (41). Interestingly, the influenza virus hemagglutinin variant with a Ser-Tyr substitution in the cleavage site was shown to be preferentially cleaved by plasmin (42). Thus, host serine protease redundancy to exert SARS-CoV-2 priming might also contribute to enhanced fitness of emerging variants.

Representative data on LFC levels in human sera are not available. However, since LFCs are digestion products derived from LF, data on serum LF levels might be correlative. According to our unpublished measurements and also published studies of others, LF levels in blood sera of healthy donors may vary within a dynamic range up to µg/mL values (43), which makes the effective concentrations around IC50 achievable.

LF and its bioactive peptides offer health benefits also beyond newborns. Cow-, goat- or sheep milk and cheese are common components of diet for adults. However, LF derived from various mammalian species may show different anti-SARS-CoV-2 abilities (29). Depiction of their inhibitory capacities against TMPRSS2 remains for future studies.

Collectively, our results reveal that not only full-length human LF but also its bioactive digestion products may be protective against COVID-19, however, *via* different mechanisms, which can produce synergic therapeutic or prophylactic effects. LF is present at high concentrations in milk and other dairy products, as well as in food supplements. Orally administered LF endows a major clinical benefit in human, particularly neonatal medicine (44), and it is considered safe and without adverse effects (45). This makes LF a cheap and widely available candidate for supplementary therapy in management of COVID-19.

## Materials and methods

### Peptides

The peptides derived from human LF were synthesized by Peptide 2.0 (Chantilly, VA). The sequences of the 19-residue synthetic peptides were as follows: GRRRSVQWCAVSQPEATKC (N-terminal pLF1, residues 1-19), EDAIWNLLRQAQEKFGKDK (middle pLF2, residues 264-282), NLKKCSTSPLLEACEFLRK (C-terminal pLF3, residues 673-691), and NFRTKSCPLELAKELKLCS (pCTR, a scrambled variant of pLF3) (11). Numbering of the LF peptides refers to a mature secreted form of the representative LF isoform P02788-1 (www.uniprot.org).

### *In vitro* proteolysis assay

The proteolytic activities of plasmin (10 nM; from human plasma; #10602361001, Roche Diagnostics GmbH, Mannheim, Germany), trypsin (10 nM; from bovine pancreas; #T1005, Sigma-Aldrich, St. Louis, MO), elastase (20 nM; from porcine pancreas; #E1250, Sigma-Aldrich) and TMPRSS2 (25 nM; recombinant protein produced in mammalian cells; #MBS1193731, MyBioSource, San Diego, CA) were measured in a Nunc black fluorescence-based cell assay plate (Sigma-Aldrich) in Tris-HCl buffer (20 mM, pH 8.0, 150 mM NaCl). The proteases were incubated with or without inhibitory and control compounds at 37°C for 30 min, and then the fluorogenic substrate (Boc-Gln-Ala-Arg-AMC; 25 µM; #BML-P237, ENZO Life Sciences, Lörrach, Germany) was added. The reaction was measured using an ELISA plate reader (Synergy H1 BioTek microplate reader, Winooski, Vermont, U.S., or TECAN GENios, GMI, USA) under the excitation wavelength of 380 nm and the emission wavelength of 460 nm at the indicated time points.

Optionally (**Figure 1B**, *inset*), purified active plasmin (10 mU/mL) was coated in PBS directly on a 96-well Falcon transparent plate for 2 h at 37°C. The wells were then blocked with 1% BSA, washed, and then incubated in PBS at 37°C with the chromogenic plasmin substrate S-2251 (0.8 mM, D-Val-Leu-Lys-pNA, CoaChrom Diagnostica). In this case, the absorbance change at 405 nm was monitored at different time points by using an ELISA plate reader (SpectraMax M5, Molecular Devices).

In the SARS-CoV-2 S protein digestion assay, purified recombinant 6x-His-tagged S protein (1 µg; #REC31868, NativeAntigen, Kidlington, UK) was exposed to TMPRSS2 (25 nM) or plasmin (10 nM) with or without inhibitory and control compounds for 120 min at 37°C in Tris-HCl buffer (20 mM, pH 7.5, 150 mM NaCl). The reaction was stopped by adding SDS-PAGE sample buffer, the reaction mixtures were then boiled and analyzed by immunoblotting with a specific anti-6x-His tag monoclonal antibody (mAb) (#631212, Clontech, Heidelberg, Germany).

### Preparation of the mutated S protein

Prefusion-stabilized SARS-CoV-2 S protein ectodomain (46) (residues 1−1208 from GenBank: MN908947), with proline substitutions at residues 986 and 987, a “GSAS” substitution at the furin cleavage site residues 682–685, a C-terminal T4 fibritin trimerization motif, an HRV3C protease cleavage site, a Twin-Strep-tag and an 8x-His tag, was expressed in CHO cells and purified as described previously (47). Briefly, the S protein secreted to cell media was captured on a 5 ml His-Trap affinity column (Cytiva, Marlborough, MA, USA), eluted with 0.5 M imidazole in 20 mM sodium phosphate buffer, pH 7.4 and further purified on the Strep-Tactin^®^ media (IBA Lifesciences GmbH, Göttingen, Germany) as described by the manufacturer, using 10 mM desthiobiotin (IBA Lifesciences) as an elution reagent. The purified S protein was concentrated by ultrafiltration and buffer-exchanged into PBS on a 5 ml HiTrap Desalting column (Cytiva). The concentration was determined by UV absorbance at 280 nm. Finally, the S protein was sterile-filtered and stored at -20°C.

### Generation of LFC and MALDI-TOF MS

Recombinant human LF (L1294, Sigma-Aldrich) was digested by pepsin (from porcine stomach mucosa; #P6887, Sigma-Aldrich). To a LF solution (10.5 mg/mL) in 150 mM NaCl, pepsin (3% or 10% per amount of LF) and HCl (10, 13, 18, 30, 41, 47, 72 or 90 mM) were added for various times (15, 30, 45 or 60 min). Upon the optimal cleavage condition (72 mM HCl and 10% pepsin for 60 min at 37°C), LF was completely cleaved into short peptides. The resulting digestion mixture was purified by cation exchange chromatography using a 1 mL Mono S column (GE Healthcare). Gradient of ammonium carbonate (20 to 1500 mM) was used for elution. Samples were analyzed by 10% tricine-SDS-PAGE and subjected to Coomassie blue staining. A fraction eluted at 73-81 mS/cm conductivity was dried and incubated at 60°C overnight to remove the ammonium carbonate. The sample was dissolved in PBS and the concentration was determined from the absorbance at 280 nm using an extinction coefficient of 11250 M^-1^cm^-1^. 40% ACN/0.1% TFA was used for dissolving the sample before MALDI analysis. Equal volume of sample and alpha-cyano-4-hydroxycinnamic acid (HCCA) matrix solution (10 mg/mL in 70% ACN/0.1% TFA) was mixed on MALDI target plate. MALDI analysis was performed in positive mode. Single and double charged ions present in spectra corresponded to a peptide with the neutral mass of 5738.1 Da, which was in good agreement with the published human LFC peptide mass (sequence GRRRRSVQWCAVSQPEATKCFQWQRNMRKVRGPPVSCIKRDSPIQCIQA) (21).

### Immunoblotting

Immunoblotting was performed as described previously (48). Briefly, samples obtained from *in vitro* proteolysis or binding assays were analyzed by electrophoresis on an 8% SDS-polyacrylamide gel (SDS-PAGE) followed by a transfer at constant voltage (15 V) to an Immobilon polyvinylidene difluoride membrane (Millipore Co., Bedford, MA). The membranes were blocked using 4% nonfat milk and immunostained with an appropriate mAb followed by a corresponding secondary HRP-conjugate. For visualization of proteins, the chemiluminescence image analyzer Azure 280 (AzureBiosystems, Dublin, CA) was used. Densitometric quantifications of corresponding bands were done by means of AzureSpot software.

### SARS-CoV-2 infection and In-Cell ELISA

The African green monkey kidney-derived Vero cells (ATCC CCL-81, provided by Prof. Sylvia Knapp, Medical University of Vienna) or human lung adenocarcinoma Calu-3 cells (ATCC HTB-55, provided by Prof. Walter Berger, Medical University of Vienna) were seeded into 96-well flat-bottom plates (1×10^4^ cells for Vero or 2×10^4^ cells for Calu-3, 80 µl/well) in Dulbecco´s Modified Eagle´s medium (DMEM), high glucose, with GlutaMAX and sodium pyruvate (Gibco/Thermo Fisher Scientific, Waltham, MA USA) supplemented with 10% fetal calf serum (FCS, Biowest, Nuaillé, France), 1% MEM non-essential amino acids solution, 100 U/mL penicillin and 100 μg/mL streptomycin (all latter from Gibco/Thermo Fisher Scientific). On the next day, tested peptides, LF and controls were diluted in DMEM medium with reduced serum (2% FCS) in a separate plate so that the concentration was 1.33-fold higher than final. Ninety µl of the solution was used to pretreat seeded cells (old medium was discarded) for approx. 40-60 min. During the incubation time, cells were transferred to the BSL3 facility of the Medical University of Vienna and then infected with 30 µl (300 TCID50/well; multiplicity of infection (MOI) ≈0.02) of the authentic SARS-CoV-2 isolate BetaCoV/Munich/BavPat1/2020 [kindly provided by Christian Drosten, Charité, Berlin (49), and distributed by the European Virology Archive (Ref-SKU: 026V-03883)] that was diluted to 1×10^4^ TCID50/mL in DMEM/2% FCS. Cells were then incubated at 37°C. After 48 h, cells were fixed by adding 45 μl/well 37% formaldehyde for 20 min. Supernatants were then aspired and the entire plate was fixed in fresh 5% formaldehyde in PBS for 30 min. Next, the formaldehyde was removed, the cells were washed with PBS, permeabilized using 0.1% Triton X-100 in PBS and blocked with blocking buffer (10% FCS in PBS+0.05% Tween-20). Subsequently, the cells were stained by indirect immunofluorescence using a rabbit anti-SARS-CoV-2 nucleocapsid mAb (40143-R019, SinoBiological, Beijing, China, diluted 1:15000 in blocking buffer) followed by a goat anti-rabbit-HRP conjugate (170-6515, Bio-Rad, Hercules, CA USA, diluted 1:10000 in blocking buffer). In-Cell ELISA was then developed using DY999 substrate solution according to the manufacturer’s recommendations (R&D Systems, Minneapolis, MN USA) and measured at 450 nm (and at 630 nm to assess the background) using a Mithras multimode plate reader (Berthold Technologies, Bad Wildbad, Germany). To calculate percent inhibition of infection in each well, the following formula was used: 100 – [(X - average of ‘no virus’ wells)/(average of ‘virus only’ wells - average of ‘no virus’wells)*100], where X is the background-subtracted read for each well, as we did previously (50, 51).

### Cytotoxicity assay

Vero cells were seeded into 96-well flat-bottom plates (1×10^4^ cells, 80 µl/well) in DMEM medium supplemented with 10% FCS, antibiotics, and non-essential amino acids. On the day of the assay, the medium was exchanged for the reduced serum supplemented DMEM (DMEM/2% FCS) with the peptides or a vehicle only at the designated concentration (100 µl/well). As a positive control, 30% DMSO in DMEM/2% FCS was used. Negative control cells were incubated in DMEM/2% FCS only. Two days (48 h) later, cell viability was assessed as we did previously (52) using the EZ4U cell proliferation and cytotoxicity assay substrate (Biomedica Immunoassays, Vienna, Austria).

### Statistical analysis

All experiments were performed at least three times in at least duplicates. The data were expressed as mean values with standard deviations (SD). Graphing and statistical significance evaluation (using a Student’s t-test or one-way ANOVA with Dunnett’s multiple comparisons post-test, unless stated otherwise) was done in Prism 9 for MacOS. Values of p*<0.05, p**<0.005, p***<0.0005, p****<0.0001 (as indicated) were considered to be significant or highly significant, respectively. Relative IC50 values were determined also in Prism by a 4PL nonlinear regression curve fit.

## Data availability statement

The original contributions presented in the study are included in the article/**Supplementary Material**. Further inquiries can be directed to the corresponding authors.

## Author contributions

VL, AO-R and RS conceived and designed the experiments. VL, GO and RP made biochemical analyses, AO-R, LG, and GT, made cell infection assays, RS, NJ, PH made chromatography purification, PB performed mass spectometry, HS contributed with ideas, comments and materials. VL wrote the paper. All authors read and corrected the manuscript. All authors contributed to the article and approved the submitted version.

## Funding

This work was supported by grants of the Science and Technology Assistance Agency of the Slovak Republic (APVV-16-0452, APVV-20-0513), of the Slovak Grant Agency VEGA (2/0020/17, 2/0152/21) and of the Austrian Science Fund (FWF; P 34253-B).

## Acknowledgments

The authors thank Prof. Sylvia Knapp, Dr. Riem Gawish and the other Knapp lab members (Medical University of Vienna, Department of Medicine I, Research Laboratory of Infection Biology, Vienna, Austria) for the support and constructive discussions. The authors also thank Prof. Walter Berger, Medical University of Vienna, Center for Cancer Research, Vienna, Austria) for providing Calu-3 cells.

## Conflict of interest

The authors declare that the research was conducted in the absence of any commercial or financial relationships that could be construed as a potential conflict of interest.

## Publisher’s note

All claims expressed in this article are solely those of the authors and do not necessarily represent those of their affiliated organizations, or those of the publisher, the editors and the reviewers. Any product that may be evaluated in this article, or claim that may be made by its manufacturer, is not guaranteed or endorsed by the publisher.

## Abbreviations

ACE2, angiotensin converting enzyme-2; Ap, aprotinin; COVID-19, coronavirus disease 2019; HSPGs, heparan sulfate proteoglycans; LF, lactoferrin; LFC, lactoferricin; mAb, monoclonal antibody; Plg, plasminogen; SARS-CoV-1, severe acute respiratory syndrome coronavirus 1; SARS-CoV-2, severe acute respiratory syndrome coronavirus 2; TMPRSS2, transmembrane protease serine 2; uPA, urokinase-type plasminogen activator.
