# Supplementary material for: Blockade of TMPRSS2-mediated priming of SARS-CoV-2 by lactoferricin
Source: Front Immunol. 2022 Aug 23;13:958581. doi: 10.3389/fimmu.2022.958581 (PMC9445877; doi:10.3389/fimmu.2022.958581)
Supplement: Supplementary file 1 [file DataSheet_1.pdf]

Supplementary Material

Blockade of TMPRSS2-mediated priming of SARS-CoV-2 by lactoferricin

Anna Ohradanova-Repic *, Rostislav Skrabana, Laura Gebetsberger, Gabor Tajti, Peter Baráth, Gabriela Ondrovičová, Romana Praženicová, Nikola Jantova, Patricia Hrasnova, Hannes Stockinger, and Vladimir Leksa*

*** Correspondence:**
Corresponding Authors
vladimir.leksa@savba.sk

anna.repic@meduniwien.ac.at

1.1 Supplementary Figures

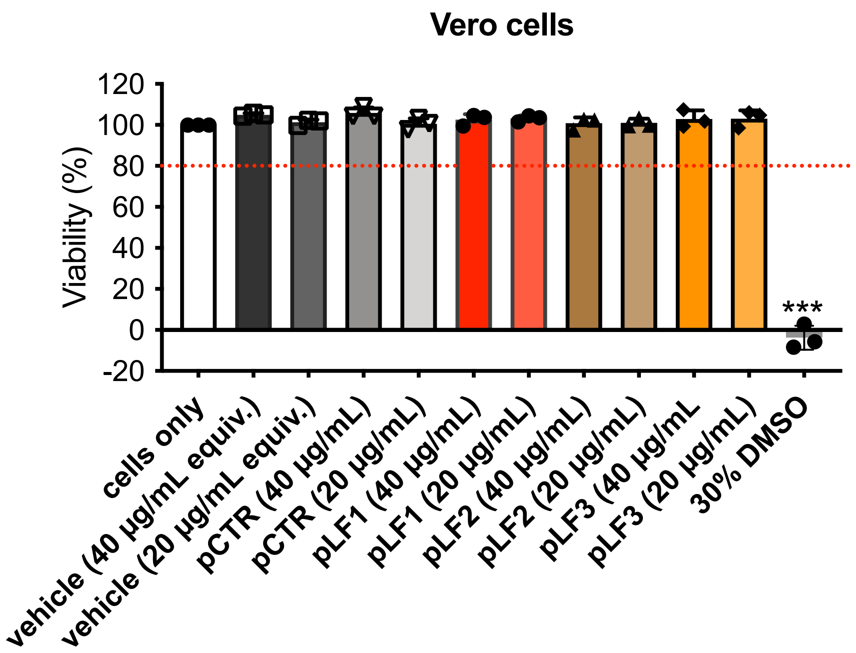

Supplementary Figure 1. To determine possible cytotoxic effects of the peptides used, viability of Vero cells was assessed after 48 h contact with different peptides or vehicle only and compared with the untreated (negative control) and 30% DMSO-treated cells (positive control). The dotted red line represents 80% viability cut-off. Data are mean ± SD of 3 experiments. Only the positive control significantly ($p^{***}<0.0005$) affected viability of Vero cells.
